# Protective effect of MOTILIPERM in varicocele-induced oxidative injury in rat testis by activating phosphorylated inositol requiring kinase 1α (p-IRE1α) and phosphorylated c-Jun N-terminal kinase (p-JNK) pathways

**DOI:** 10.1080/13880209.2017.1421672

**Published:** 2018-01-10

**Authors:** Kiran Kumar Soni, Li Tao Zhang, Bo Ram Choi, Keshab Kumar Karna, Jae Hyung You, Yu Seob Shin, Sung Won Lee, Chul Young Kim, Chen Zhao, Han-Jung Chae, Hye Kyung Kim, Jong Kwan Park

**Affiliations:** a Department of Urology, Chonbuk National University and Research Institute of Clinical Medicine of Chonbuk National University-Biomedical Research Institute and Clinical Trial Center of Medical Device of Chonbuk National University, Jeonju, Republic of Korea;; b Department of Urology, Samsung Medical Center, Samsung Biomedical Research Institute, Sungkyunkwan University School of Medicine, Seoul, Republic of Korea;; c College of Pharmacy, Hanyang University, Ansan, Republic of Korea;; d Department of Urology, Renji Hospital, Shanghai Jiao Tong University School of Medicine, and Shanghai Institute of Andrology, Shanghai, China;; e Department of Pharmacology, Chonbuk University of Medical School, Jeonju, Republic of Korea;; f College of Pharmacy, Kyungsung University, Busan, Republic of Korea

**Keywords:** Sperm, oxidative stress, ER stress, testosterone

## Abstract

**Context:** MOTILIPERM was prepared as a mixture of extracts of three medicinal herbs [roots of *Morinda officinalis* How (Rubiaceae), outer scales of *Allium cepa* L. (Liliaceae) and seeds of C*uscuta chinensis* Lamark (Convolvulaceae)].

**Objective:** To investigate the role of reactive oxygen species (ROS)-based endoplasmic reticulum (ER) stress in a rat model of varicocele and the therapeutic efficacy of MOTILIPERM in this model.

**Materials and methods:** Sixty male rats were divided into five experimental groups: a normal control group (CTR + vehicle), a control group administered MOTILIPERM 200 mg/kg (CTR + M 200), a varicocele-induced control group (VC + vehicle) and two varicocele-induced groups administered MOTILIPERM 100 (VC + M 100) or 200 (VC + M 200) mg/kg for 4 weeks. Testis weights were recorded and serums were assayed for hormone concentrations. Tissues were subjected to semen analysis, histopathology, analyses of ER response protein expression levels and oxidative stress were assessed by measuring ROS, reactive nitrogen species (RNS), malondialdehyde (MDA) level and ratios of total glutathione (GSH)/oxidized GSH (GSSG).

**Results:** MOTILIPERM treatment of varicocele-induced groups significantly increased left testis weight, testosterone level, sperm motility, count and spermatogenic cell density. ER-response protein expression levels were dose-dependently decreased in VC + M 200 group compared with VC + vehicle group. MOTILIPERM treatment also decreased MDA and ROS/RNS level but increased GSH/GSSG ratio.

**Discussion and conclusions:** This study suggests that ROS-related ER stress may play a major role in varicocele-induced infertility and MOTILIPERM, a novel compound targeting ROS-based ER stress, may be therapeutically useful in treatment of varicocele, or as a supplement for the treatment of infertility.

## Introduction

Infertility is a disease of the reproductive system defined by the failure to non-contracepting couple to conceive after 1 year of regular intercourse (Jung et al. 2016). Infertility is an important public health issue, affecting 13–15% of couples of reproductive age (Jarow et al. 2002). About 40–50% of all cases are related to male infertility (Kantartzi et al. 2007). Different factors can compromise male fertility potential; including congenital and genetic abnormalities, exposure to gonadotoxins, genitourinary infections, immunologic factors, endocrine disorders, systemic diseases and cancer. Varicocele accounts for 35% of all cases of male infertility (Madgar et al. 1995). Varicocele is a condition associated with abnormal tortuosity and dilatation of the veins of the pampiniform plexus within the spermatic cord (Zhang et al. 2014). Reports relating to the presence of varicocele in testis date back to the first century A.D. by Greek physician Celsus (Saypol 1981). The possible link between varicocele and infertility was first noticed between the end of the 19th and the beginning of the twentieth century when surgical repair of varicocele improved the quality of sperm (Tulloch 1951). Varicoceles are a correctable cause of male infertility and 40% of men with primary infertility (Chiba and Fujisawa 2016). Varicocele can affect spermatogenesis in many ways e.g., increased testicular temperature, increased intratesticular pressure, hypoxia due to attenuation of blood flow, reflux of toxic metabolites from the adrenal glands and hormonal profile abnormalities (Wright et al. 1997). Varicocele also has been associated with increased oxidative stress, especially in the gonads (Allamaneni et al. 2004).

Reactive oxygen species (ROS) are byproducts of metabolism and are harmful in a variety of ways (Agarwal et al. 2012). Accumulation of free radicals has been implicated in the pathogenesis of several disease states and several markers are indicative of oxidative stress. Glutathione disulphide (GSSG) is the oxidized form of glutathione (GSH) and the ratio of one to the other reflects the extent of oxidative stress. Malondialdehyde (MDA) is a low molecular weight end product of lipid hydroperoxide decomposition and is the most frequently measured index of lipid peroxidation. Changes in testicular haemodynamics due to varicocele probably increases ROS (Nallella et al. 2004). Increased levels of oxidative stress markers are found in serum, semen and testicular tissues of varicocele patients (Agarwal et al. 2005). It has been hypothesized that the presence of varicocele in males is associated with progressive testicular damage from adolescence and results in diminished fertility (Jungwirth et al. 2012). In addition, varicocele is associated with increased sperm DNA damage, possibly secondary to varicocele mediated oxidative stress. This ultimately decreases semen quality and sperm function (Zini and Dohle 2011). Oxidative stress manifests when ROS overcome the natural antioxidant defences of semen and damage sperm (Tremellen 2008). So, natural intracellular and extracellular antioxidants scavenge and neutralize the harmful effects of ROS (Agarwal et al. 2012).

ROS are crucial regulators of endoplasmic reticulum (ER) function and activation of the unfolded protein response (UPR) in disease conditions. Thus, ER stress and increased ROS production occur concurrently (Cao and Kaufman 2014; Zeeshan et al. 2016). Antioxidants are widely available and inexpensive when compared to other fertility treatments and are used by many men to improve fertility. Oral antioxidant supplements may improve sperm quality by reducing oxidative stress (Lombardo et al. 2011).

MOTILIPERM was prepared as a mixture of natural extracts of three medicinal herbs: roots of *Morinda officinalis* How (Rubiaceae), outer scales of *Allium cepa* L. (Liliaceae), and seeds of C*uscuta chinensis* Lamark (Convolvulaceae). The root of *Morinda officinalis* is used to treat rheumatoid arthritis and impotence in traditional oriental medicine. Monotropein and deacetyl asperulosidic acid are the major iridoid compounds in *Morinda officinalis*. Monotropein was shown to exhibit antinociceptive and anti-inflammatory activities (Choi et al. 2005; Shin et al. 2013). Hyperoside and kaempferol 3-*O*-glucoside are major flavonoids of the seeds of *Cuscuta chinensis*, which is an important oriental traditional medicine widely used to improve sexual function, and prevent and treat cardiovascular diseases (Löffler et al. 1997; Yang et al. 2011). The outer scales of onion contain large amounts of quercetin and quercetin 4′-*O*-glucoside, those are effective antioxidants. Other pharmacologically useful activities, such as liver protection, immune enhancement, anti-infection, anti-stress and anti-cancer effects were also reported (Singh et al. 2009). MOTILIPERM is a novel compound that acts as an antioxidant and is under development for the treatment of male infertility. However, no data are available on the effects of MOTILIPERM on ROS-based ER stress in varicocele-related infertility. Therefore, our aim was to further investigate the mechanism underlying development of male infertility caused by varicocele and to assess the effect of MOTILIPERM in rats with experimentally induced varicocele.

## Materials and methods

### Animals and experimental protocol

All animal experiments in this study were performed in accordance with the Guide for the Care and Use of Laboratory Animals of Chonbuk National University and were approved by the Institutional Animal Care and Use Committee of Chonbuk National University Laboratory Animal Center (Cuh-IACUC-1507-27). Sixty sexually mature male Sprague-Dawley rats (300–320 g, 10–12 weeks of age) were fed standard rat chow and had free access to water. They were maintained in an animal facility under constant environmental conditions (room temperature, 20 ± 2 °C; relative humidity, 50 ± 10%; and a 12 h light–dark cycle). All rats were placed in cages and fed after they were operated on.

Rats were divided into five groups of 12: CTR + vehicle, a normal control group; CTR + M 200, normal rats administered 200 mg/kg MOTILIPERM by oral gavage; VC + vehicle, the varicocele-induced controls; VC + M 100, varicocele-induced rats given 100 mg/kg MOTILIPERM by oral gavage and VC + M 200, varicocele-induced rats administered 200 mg/kg MOTILIPERM by oral gavage. MOTILIPERM administration was performed daily for 4 weeks commencing 4 weeks after the varicocele induction. At 8 weeks after varicocele induction surgery, rats were anesthetized, blood samples were collected for testosterone analyses and testis tissues were collected. Testes were assayed for ROS/reactive nitrogen species (RNS), MDA levels, total (GSH) activity, oxidized GSH (GSSG) activity, pathological histology and ER stress according to the procedures described below.

### Varicocele induction

Rats were anesthetized with a mixture of ketamine (100 mg/mL) and 2% xylazine hydrochloride (20 mg/mL). The mixture (170–230 μL/100 g body weight) was administered intramuscularly on the side of the back paw. According to veterinary anaesthesia and analgesia, stage III anaesthesia was determined by the presence of unconsciousness with progressive depression of reflexes. Muscular relaxation developed in the rats and ventilation slowed but remained regular. The vomiting and swallowing reflexes were also lost.

Branches of the left renal vein and the left internal spermatic vein (ISV) that communicate with the common iliac vein were partially ligated, and the communicating branches along the left ISV were fully ligated, as described previously (Zhang et al. 2014). In brief, the upper left abdominal quadrant was approached, and the left renal and ISV were located through a midline abdominal incision. The left renal vein was cleared of adhering tissue by blunt dissection at a position medial to insertion of the ISV and was ligated with a 4-0 black silk suture with a metal probe (outer diameter, 0.85 mm). The branch of the left ISV that communicates with the common iliac vein was ligated with a 4-0 black silk suture with a metal probe (outer diameter, 0.85 mm). The metal probe was withdrawn after ligation.

### Plant material and HPLC analysis of MOTILIPERM

MOTILIPERM is under development by the Dong-A Pharmaceutical Company (Kyunggi, South Korea) for the treatment of infertility. This extract was dissolved in sterile normal saline and administrated orally with a Zonde needle (JD-S-124, Jeungdo, Seoul, Korea) at a dose of 0 (vehicle), 100 or 200 mg/kg. The volumes of administration were 6 mL/kg delivered orally.

**Figure 1.**
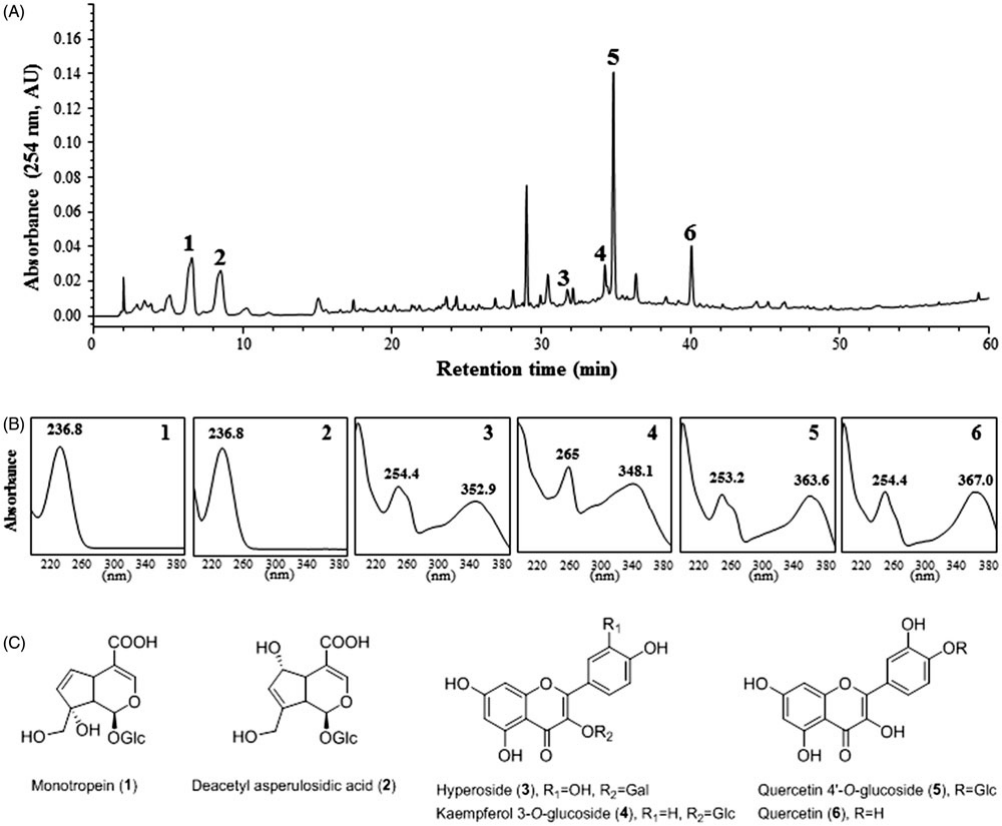
HPLC chromatogram of MOTILIPERM and UV spectra of major marker components of herbal ingredients. (A) HPLC chromatogram. (B) UV spectra. (C) Chemical structures. Monotropein (1) and deacetyl asperulosidic acid (2) in *Morinda officinalis*, hyperoside (3) and kaempferol 3-*O*-glucoside (4) in the seeds of *Cuscuta chinensis*, quercetin 4-*O*-glucoside (5) and quercetin (6) in *Allium cepa*. Each peak of MOTILIPERM in the HPLC chromatogram was identified by comparison with retention times and UV spectra of standard compounds.

Herbs were ground and extracted with ethanol under reflux for 3 h at 70–80 °C three times. The combined filtrate was concentrated in a rotary evaporator, freeze-dried, and then stored at –20 °C until required. The quality of MOTILIPERM was determined using high-performance liquid chromatography (HPLC). Twenty milligrams of MOTILIPERM was dissolved in 30 mL of methanol and filtered through a 0.45 μm membrane filter. Filtrate (10 μL) was injected into the HPLC system for analysis. HPLC peaks were identified by comparison with the retention times and UV spectra of standard compounds. The HPLC profile of MOTILIPERM and its identified components are shown in Figure 1. Quality control of MOTILIPERM as a mixture of herbal extracts requires marker compounds. Peak identification was performed by comparison of HPLC retention times and UV spectra of purified standard compounds. Monotropein (1) and deacetyl asperulosidic acid (2) in *Morinda officinalis*; hyperoside (3) and kaempferol 3-*O*-glucoside (4) in the seeds of *Cuscuta chinensis*; quercetin 4′-*O*-glucoside (5) and quercetin (6) in *Allium cepa* were purified by repeated chromatography and preparative HPLC (Figure 1). Their chemical structures were determined by NMR and compared with previously reported data (Choi et al. 2005; Ly et al. 2005; Lee et al. 2010).

### Dose calculation

MOTILIPERM dose was calculated using an FDA-approved method for medications under development (Food Drug Administration 2005). The dose of this medication for humans is 3 g/60 kg/day. The rat-equivalent dose (RED) was based on the human dose multiplied by 0.16. For example, if the human dose is 3000 mg/60 kg/day, the converted dosage for rats is 3000 mg/60 kg/day ×0.16 = 312.5 mg/kg/day. However, 300 mg/kg/day MOTILIPERM did not improve sperm count any more than 200 mg/kg/day in a previous preclinical *in vivo* study. Thus, the maximum dose used in this study was 200 mg/kg/day.

### Haematology, serum biochemistry and hormone assays

Blood was obtained from the abdominal vein. Immediately after blood collection, the CBC of the anticoagulated blood samples was measured (Vet ABC, Heska, Loveland, CO). Organs such as the testis, epididymis and seminal vesicles were surgically removed. One piece of testis tissue was collected from the same position in every rat and fixed with Bouin’s solution for histopathological analyses. Serum samples were obtained after venous blood collection and centrifugation at 3000 rpm for 15 min. Both serum aspartate aminotransferase (AST) and alanine aminotransferase (ALT) activities were measured according to the International Federation of Clinical Chemistry reference method (ASAN Pharmaceutical Co., Ltd, Seoul, Korea). Serum levels of testosterone were measured using a commercial kit (TESTO-CT2 Kit, Cisbio Inc., Bedford, MA). All protocols were performed according to the manufacturer’s instructions.

### Sperm motility and sperm counts in the vas deferens and epididymis

The epididymis and vas deferens were removed and placed in separate 1.5 mL microcentrifuge tubes, minced and suspended in normal saline at 37 °C for 5 min. Sperm motility was evaluated by observing the sperm suspension within 3–5 min after being placed in the counting chamber (Makler/Sperm Meter, Product Code: Sperm Counting Chamber; SEFI-Medical Instruments Ltd, New York, NY). This method mitigates error due to the tendency of spermatozoa to migrate from the periphery. The number of motile spermatozoa within the 10 central squares of the grid were counted under an Axio Imager 2 light microscope (Carl Zeiss MicroImaging LLC, Goettingen, Germany) and mean sperm counts were recorded. The percentage of motile spermatozoa was determined by the following formula: (mean number of motile spermatozoa/total number of spermatozoa) × 100.

The total sperm count was calculated using two or three drops of each specimen to improve the reliability of the count. The counting chamber is composed of two parts: The upper part is a cover glass encircled with a metal ring. At the centre of its lower surface, there is a 1 mm^2^ grid that is subdivided into 100 squares of 0.1 × 0.1 mm. When the cover glass is placed on the four tips, the volume bounded in a row of 10 squares is exactly one-millionth of 1 mL. Therefore, the number of sperm heads in 10 squares indicates their concentration in million/mL and the mean value is reported. Spermatozoa were counted using the 20× objective on the microscope.

### Spermatogenic cell density

Testis tissues were collected to assess the effects of MOTILIPERM on pathological changes. Testes were fixed in Bouin’s solution (Sigma-Aldrich, St. Louis, MO) for 4–6 h and washed with 70% alcohol. The tissue samples were embedded in paraffin and then 5 μm sections were cut, deparaffinized, rehydrated and stained with haematoxylin and eosin (H&E). Four consecutive sets of 10 sections were used. Of these two sets were stained with H&E. Testis tissue was evaluated using standard light microscopy. Three nearly round seminiferous tubules were assessed in each section, according to the presence of spermatogenic cells. Spermatogenic cell density was determined by measuring the thickness of the germinal cell layer and the diameter of the seminiferous tubules.

### Johnsen’s score

The seminiferous tubules were graded according to the Johnsen scoring system. In this method, 20 seminiferous tubules are assessed according to the presence of spermatogenic cells, and each is assigned a score from 1 to 10. Complete spermatogenesis with many spermatozoa present is given a score of 10. The detailed histological criteria for scoring are 1: no seminiferous epithelium; 2: no germinal cells (Sertoli cells only); 3: spermatogonia only; 4: no spermatozoa or spermatids, with few spermatocytes; 5: no spermatozoa or spermatids, with many spermatocytes; 6: no spermatozoa, no late spermatids and few early spermatids; 7: no spermatozoa, no late spermatids and many early spermatids; 8: fewer than five spermatozoa per tubule, and few late spermatids; 9: slightly impaired spermatogenesis, many late spermatids, and a disorganized epithelium; and 10: full spermatogenesis (Zhang et al. 2014).

### Western blotting

Glucose-regulated protein-78 (GRP-78), phosphorylated inositol requiring transmembrane kinase/endoribonuclease 1α (p-IRE1α) and phosphorylated c-Jun-N-terminal kinase (p-JNK) levels were determined using testis tissue that had been washed with cold PBS. Lysis buffer with protease inhibitor was added to the tissue and then cordless motor pellet pestles were used to grind the tissue (Sigma-Aldrich, St. Louis, MO), before sample centrifugation at 12,000 rpm for 30 min at 4 °C.

The samples were run on 10% sodium dodecyl sulphate (SDS) gels, and transferred to polyvinylidene fluoride (PVDF) membranes using a Trans-blot^®^ SD semi-dry electrophoretic transfer cell (Bio-Rad, Hercules, CA). After transfer, the membranes were blocked with 10% bovine serum albumin (BSA) for 1 h and incubated overnight at 4 °C with phosphorylated antibodies 1:1000 dilution against p-IRE1α (Abcam Cambridge, MA) and p-JNK (Santa Cruz Biotechnology, Santa Cruz, CA). Non-fat milk (10%) was used for non-phosphorylated antibodies against GRP-78 and β-actin (Santa Cruz Biotechnology, Santa Cruz, CA). The membranes were washed with tris-buffered saline tween 20 (TBST) three times prior to the addition of a 1:5000 dilution of secondary antibody and incubation for 1 h. The membranes were again washed three times with TBST and developed using an enhanced chemiluminescence substrate. The bands were quantified by ImageJ software (National Institutes of Health, Bethesda, MD).

### ROS/RNS and MDA levels

The ROS/RNS assay was performed using a fluorescence kit (Cell Biolabs, Inc., San Diego, CA) at excitation and emission wavelengths of 480 and 530 nm, respectively, with a SpectraMax Gemini XS Fluorometer. MDA was assayed using spectrophotometric methods (Northwest Life Science, Ottawa, ONT, Canada) based on the reaction of MDA with thiobarbituric acid (TBA), which forms an MDA-TBA2 complex that absorbs at 532 nm.

### Total GSH and GSSG activity

GSH activity was measured immediately using a GSH colorimetric assay (Northwest Life Science, Vancouver, Canada). Supernatants were collected and assayed as per the manufacturer’s instructions. The deproteinized supernatant was further incubated with 4-vivinylpyridine, a thiol-blocking agent, for 60 min at room temperature to scavenge free GSH in the samples in the GSSG assay. Supernatants were mixed with 5,5′-dithiobis (2-nitrobenzoic acid), NADPH and GSH reductase. Samples were incubated in the dark for 3 min, and total GSH was quantified using kinetic spectrophotometric analysis at 405 nm with a Spectra Max 180 (Molecular Devices, Sunnyvale, CA). GSH concentrations were calculated from the appropriate calibration curves. Free GSH concentration was obtained by subtracting GSSG from total GSH.

### Statistical analysis

Results are expressed as mean ± SD. The statistical significance of differences was calculated by one-way analysis of variance (ANOVA), followed by Bonferroni’s multiple comparison test. *p* Values <0.05 were considered statistically significant.

## Results

### Changes in body weight and testis weights

Initial body weight, final body weight and right testis weight showed no significant changes between groups. Left testis weights were significantly increased in the VC + M 100 and VC + M 200 compared to VC + vehicle group (Table 1).

**Table 1.** The effect of the MOTILIPERM on weights of body and testis.

	CTR + vehicle	CTR + M 200	VC + vehicle	VC + M 100	VC + M 200
Initial body weight (g)	358.25 ± 18.23	348.58 ± 25.67	342.44 ± 19.80	334.55 ± 21.22	335.27 ± 36.05
Final body weight (g)	434.25 ± 30.62	409.83 ± 33.25	433.67 ± 25.08	406.27 ± 29.84	409.18 ± 34.10
Right testis (g)	2.13 ± 0.13	2.11 ± 0.20	2.07 ± 0.10	1.98 ± 0.10	2.01 ± 0.15
Left testis (g)	2.15 ± 0.13*	2.10 ± 0.17*,#	1.64 ± 0.37	1.91 ± 0.11*	1.92 ± 0.20*

Data are presented in mean ± SD. CTR + vehicle: normal control group; CTR + M 200: normal rats administered 200 mg/kg MOTILIPERM; VC + vehicle: varicocele-induced rats; VC + M 100: varicocele-induced rats administered 100 mg/kg MOTILIPERM; VC + M 200: varicocele-induced rats administered 200 mg/kg MOTILIPERM.

# Significantly different from CTR + vehicle group (*p* < 0.05).

* Significantly different from VC + vehicle group (*p* < 0.05).

### Effects of MOTILIPERM on serum biochemical markers and testosterone

Testosterone levels in the VC + vehicle group were significantly lower than the CTR + vehicle group (*p* < 0.05), whereas they were significantly higher in the VC + M 200 group compared to VC + vehicle group (*p* < 0.05) (Table 2). The values of white blood cells (WBC), red blood cells (RBC), haemoglobin (Hb), haematocrit (Hct), AST and ALT in the CTR groups and varicocele-induced groups were similar at all treatment doses.

**Table 2.** The effect of the MOTILIPERM on serum biochemical markers and testosterone level.

	CTR + vehicle	CTR + M 200	VC + vehicle	VC + M 100	VC + M 200
Testosterone (ng/mL)	3.01 ± 1.16	3.08 ± 1.66	1.66 ± 1.33#	2.02 ± 0.5	2.5 ± 1.75*
WBC (×10^3^/μL)	7.10 ± 1.35	6.94 ± 1.40	7.69 ± 1.45	7.65 ± 0.72	7.20 ± 2.78
RBC (×10^4^/μL)	8.12 ± 0.46	8.12 ± 0.55	8.44 ± 0.33	8.46 ± 0.16	8.13 ± 0.39
Hb (g/dL)	14.80 ± 1.00	14.34 ± 0.87	14.00 ± 0.39	13.97 ± 0.96	13.88 ± 0.57
Hct (%)	43.43 ± 3.10	43.42 ± 1.59	43.21 ± 2.72	41.46 ± 2.40	41.79 ± 1.56
AST (IU/L)	110.67 ± 34.02	100.25 ± 16.55	117.22 ± 28.83	108.27 ± 28.87	97.55 ± 22.36
ALT (IU/L)	67.75 ± 21.00	51.83 ± 9.49	55.22 ± 11.30	58.00 ± 20.79	49.55 ± 8.15

WBC: white blood cell; RBC: red blood cell; Hb: haemoglobin; Hct: haematocrit; AST: aspartate aminotransferase; ALT: alanine aminotransferase; CTR + vehicle: normal control group; CTR + M 200: normal rats administered 200 mg/kg MOTILIPERM; VC + vehicle: varicocele-induced rats; VC + M 100: varicocele-induced rats administered 100 mg/kg MOTILIPERM; VC + M 200: varicocele-induced rats administered 200 mg/kg MOTILIPERM.

Data are presented in mean ± SD.

# Significantly different from CTR + vehicle group (*p* < 0.05).

* Significantly different from VC + vehicle group (*p* < 0.05).

### Effects of MOTILIPERM on sperm count and motility

Mean sperm counts and motility in vas deferens and epididymis are shown in Table 3. Sperm count and motility in the VC + vehicle group were significantly lower than those in the CTR + vehicle group (*p* < 0.05), whereas sperm count and motility were significantly higher in the VC + M 200 group compared with the VC + vehicle group (*p* < 0.05).

**Table 3.** The effect of the MOTILIPERM on sperm motility and count of vas deferens, epididymis.

	CTR + vehicle	CTR + M 200	VC + vehicle	VC + M 100	VC + M 200
Motility (%)					
Vas deferens	58.60 ± 08.57	55.72 ± 04.28	27.15 ± 17.14#	45.70 ± 17.14	48.57 ± 08.57*
Epididymis	51.42 ± 05.72	51.42 ± 04.29	25.71 ± 11.43#	40.10 ± 19.90	47.14 ± 11.42*
Count (10^6^/mL)					
Vas deferens	24.28 ± 02.86	25.43 ± 07.14	14.28 ± 06.42#	21.42 ± 12.14	25.00 ± 05.71*
Epididymis	41.66 ± 03.33	40.83 ± 02.91	25.00 ± 05.83#	30.83 ± 09.16	43.33 ± 06.66*

CTR + vehicle: normal control group; CTR + M 200: normal rats administered 200 mg/kg MOTILIPERM; VC + vehicle: varicocele-induced rats; VC + M 100: varicocele-induced rats administered 100 mg/kg MOTILIPERM; VC + M 200: varicocele-induced rats administered 200 mg/kg MOTILIPERM.

Data are presented in mean ± SD.

# Significantly different from CTR + vehicle group (*p* < 0.05).

* Significantly different from VC + vehicle group (*p* < 0.05).

### Effects of MOTILIPERM on the varicocele-induced GSH/GSSG ratio, MDA and ROS/RNS levels

The GSH/GSSG ratio was significantly lower in varicocele-induced groups, with or without MOTILIPERM (VC + vehicle group, 11.81 ± 3.63; VC + M 100 group, 15.45 ± 5.45; and VC + M 200 group, 26.36 ± 4.54) compared with that in the CTR + vehicle group (44.54 ± 10.9) and the CTR + M 200 group (43.63 ± 11.72) (Table 4). The GSH/GSSG ratios after treatment were dose-dependently higher in the VC + M 100 group and VC + M 200 group compared with the VC + vehicle group.

**Table 4.** The effect of the MOTILIPERM on varicocele induced oxidative stress.

	CTR + vehicle	CTR + M 200	VC + vehicle	VC + M 100	VC + M 200
GSH/GSSG ratio	44.54 ± 10.90	43.63 ± 11.72	11.81 ± 3.63#	15.45 ± 5.45	26.36 ± 4.54*
MDA (nmol/mg)	31.25 ± 0.63	31.87 ± 0.75	37.50 ± 2.50#	35.00 ± 1.88*	30.63 ± 1.88*
ROS/RNS (nmol/µg)	4.41 ± 0.16	4.71 ± 0.18	5.42 ± 0.40#	5.37 ± 0.31#	4.55 ± 1.05

ROS: reactive oxygen species; RNS: reactive nitrogen species; MDA: malondialdehyde; GSH: glutathione; GSSG: glutathione disulfide; CTR + vehicle: normal control group; CTR + M 200: normal rats administered 200 mg/kg MOTILIPERM; VC + vehicle: varicocele-induced rats; VC + M 100: varicocele-induced rats administered 100 mg/kg MOTILIPERM; VC + M 200: varicocele-induced rats administered 200 mg/kg MOTILIPERM.

Data are presented in mean ± SD.

# Significantly different from CTR + vehicle group (*p* < 0.05).

* Significantly different from VC + vehicle group (*p* < 0.05).

The VC + vehicle group showed higher MDA levels than the CTR + vehicle group (*p* < 0.05) (Table 4). Furthermore, the VC + M 200 group displayed significantly lowered MDA levels (*p* < 0.05) compared to VC + vehicle group. These results show that MOTILIPERM can reduce peroxidation induced by varicocele.

ROS/RNS levels in testis were significantly higher in the VC + vehicle group and VC + M 100 group compared with the CTR + vehicle group (*p* < 0.05) (Table 4).

### Effects of MOTILIPERM on varicocele-induced histological changes in the testis

**Figure 2.**
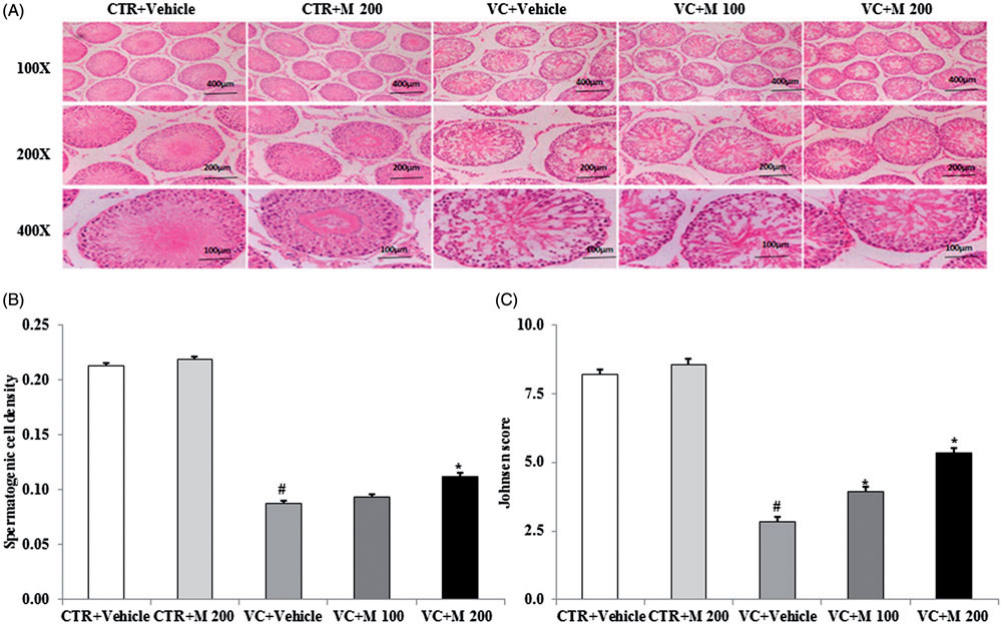
Histologic findings in testis from control groups and varicocele-induced groups. (A) Histological changes in the testis. (B) Spermatogenic cell density. (C) Johnsen’s score of the seminiferous tubules. Original magnifications 100×, 200× and 400×. CTR + vehicle: normal control group; CTR + M 200: normal rats administered 200 mg/kg MOTILIPERM; VC + vehicle: varicocele-induced rats; VC + M 100: varicocele-induced rats administered 100 mg/kg MOTILIPERM; VC + M 200: varicocele-induced rats administered 200 mg/kg MOTILIPERM. #Significantly different from CTR + vehicle group (*p* < 0.05). *Significantly different from VC + vehicle group (*p* < 0.05).

H&E staining of testis sections showed that the organization of the seminiferous epithelium or the layer of spermatogenic cells was irregular and the lumens were narrowed in varicocele-induced groups compared with control groups (Figure 2(A)). In addition, the numbers of spermatogonia and spermatocytes were significantly reduced in the varicocele with or without MOTILIPERM groups compared to the CTR + vehicle and CTR + M 200 groups. No histological abnormalities were observed in the CTR + vehicle or CTR + M 200 group.

H&E staining showed enhanced spermatogenic cell density in the VC + M 200 group compared with the VC + vehicle group (*p* < 0.05) (Figure 2(B)). Significant changes in the seminiferous tubules were observed both in the VC + M 100 group and VC + M 200 group compared with VC + vehicle group, indicating that varicocele-induced damage in the seminiferous tubules was ameliorated by MOTILIPERM (*p* < 0.05) (Figure 2(C)). In particular, a significant increase in the number of spermatogenic cells was observed with 200 mg/kg MOTILIPERM.

### MOTILIPERM attenuates ROS-mediated ER stress in the testis

**Figure 3.**
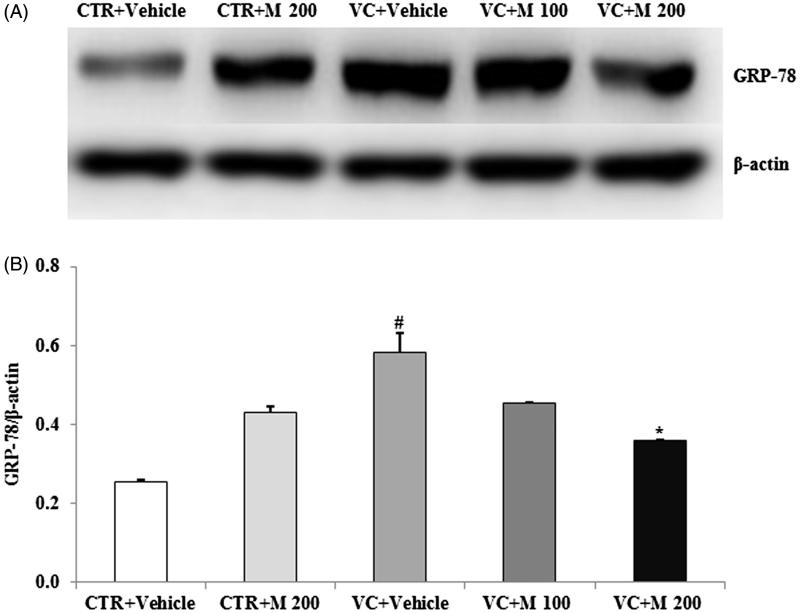
Effects of MOTILIPERM on levels of GRP-78 protein in the varicocele-induced endoplasmic reticulum stress response. (A) Western blot of testis. (B) Level of GRP-78 protein for each group. Beta-actin used as a loading control to normalize the GRP-78 protein levels in each sample. GRP-78: glucose-regulated protein-78; CTR + vehicle: normal control group; CTR + M 200: normal rats administered 200 mg/kg MOTILIPERM; VC + vehicle: varicocele-induced rats; VC + M 100: varicocele-induced rats administered 100 mg/kg MOTILIPERM; VC + M 200: varicocele-induced rats administered 200 mg/kg MOTILIPERM. #Significantly different from CTR + vehicle group (*p* < 0.05). *Significantly different from VC + vehicle group (*p* < 0.05).

The levels of the main proteins related to ER stress were affected by varicocele. Among the levels of ER stress-related molecules GRP-78, p-IRE1α and p-JNK, the GRP-78 level was significantly higher in the VC + vehicle group compared to the CTR + vehicle group. Moreover, the GRP-78 level was significantly lower in the VC + M 200 group compared to the VC + vehicle group (Figure 3).

**Figure 4.**
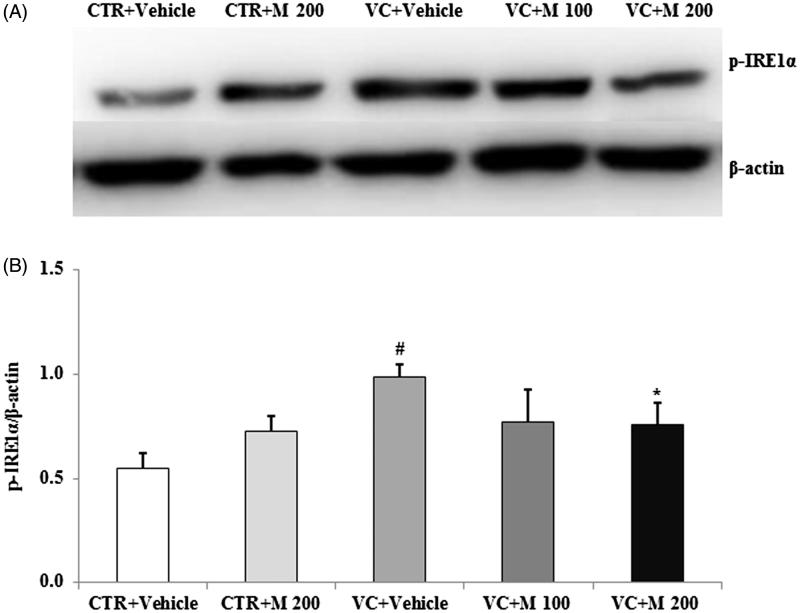
Effects of MOTILIPERM on the level of p-IRE1α protein in the varicocele-induced endoplasmic reticulum stress response. (A) Western blot of testis. (B) Level of p-IRE1α protein for each group. Beta-actin used as a loading control to normalize the p-IRE1α protein levels in each sample. p-IRE1α: phosphorylated inositol requiring transmembrane kinase/endoribonuclease 1α; CTR + vehicle: normal control group; CTR + M 200: normal rats administered 200 mg/kg MOTILIPERM; VC + vehicle: varicocele-induced rats; VC + M 100: varicocele-induced rats administered 100 mg/kg MOTILIPERM; VC + M 200: varicocele-induced rats administered 200 mg/kg MOTILIPERM. #Significantly different from CTR + vehicle group (*p* < 0.05). *Significantly different from VC + vehicle group (*p* < 0.05).

There was a significant increase of p-IRE1α expression level in VC + vehicle group compared with CTR + vehicle group (Figure 4). Significantly lower levels of p-IRE1α were seen in VC + M 200 group (0.76 ± 0.11) compared with CTR + vehicle group (0.98 ± 0.06).

**Figure 5.**
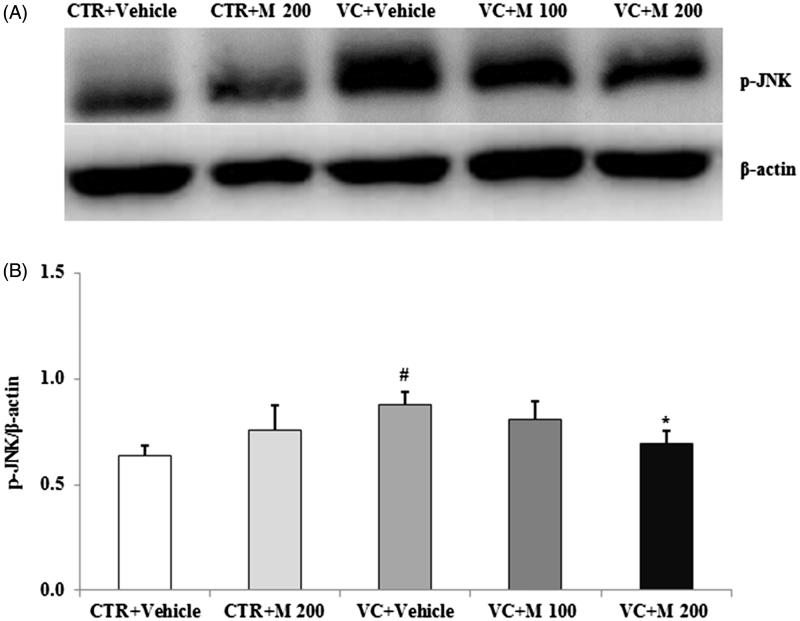
Effects of MOTILIPERM on the level of p-JNK protein in the varicocele-induced endoplasmic reticulum stress response. (A) Western blot of testis. (B) Level of p-JNK protein for each group. Beta-actin used as a loading control to normalize the p-JNK protein levels in each sample. p-JNK: phosphorylated c-Jun-N-terminal kinase; CTR + vehicle: normal control group; CTR + M 200: normal rats administered 200 mg/kg MOTILIPERM; VC + vehicle: varicocele-induced rats; VC + M 100: varicocele-induced rats administered 100 mg/kg MOTILIPERM; VC + M 200: varicocele-induced rats administered 200 mg/kg MOTILIPERM. #Significantly different from CTR + vehicle group (*p* < 0.05). *Significantly different from VC + vehicle group (*p* < 0.05).

The p-JNK level was significantly increased in VC + vehicle group (0.88 ± 0.06) compared to CTR + vehicle group (0.64 ± 0.05) and it was decreased in the VC + M 200 group (0.69 ± 0.06) compared with the VC + vehicle group (Figure 5).

## Discussion

Varicocele is one of the reasons for male fertility (Kantartzi et al. 2007; Agarwal et al. 2012). Infertility in varicocele is due to reduced semen parameters, testicular size and testosterone levels (Miyaoka and Esteves 2012; Zhang et al. 2014). In this study, we found that sperm count and sperm motility of the vas deferens and epididymis, testosterone levels and left testis weights were significantly reduced in VC + vehicle group. Johnsen’s score and spermatogenic cell density were also reduced in VC + vehicle group. Varicocele induced oxidative stress markers in rat testis. ROS/RNS and MDA were increased whereas GSH/GSSH was reduced in the testis tissue with varicocele-induced oxidative stress. There are higher levels of seminal ROS and lipid peroxidation in infertile men either with or without varicocele men than fertile men (Inci and Gunay 2013). Impairments in seminal antioxidant and lipid peroxidation status play important roles in the physiopathology of male infertility (Collodel et al. 2015). Seminal plasma MDA level has been shown to be negatively correlated with sperm viability (Das et al. 2009), sperm motility, sperm morphology and sperm concentration (Benedetti et al. 2012), whereas the MDA level is positively correlated with acrosome anomalies and the presence of residual cytoplasmic droplets (Collodel et al. 2015). Infertile males with varicocele have been reported to have higher MDA levels than do fertile males (Tawadrous et al. 2013). In one study, the micronized purified flavonoid fraction displayed antioxidant activity and inhibited MDA (Dogan et al. 2014). MOTILIPERM was used as an antioxidant to reduce the oxidative stress induced by varicocele. Therefore, measuring ROS and other markers of oxidative stress, including the levels of antioxidant enzymes, provides valuable information on the extent of oxidative stress and could help guide therapeutic management strategies.

The relationship between oxidative stress and ER stress is bidirectional, and the accumulation of unfolded proteins in the ER lumen is sufficient to trigger production of ROS (Kaufman et al. 2010; Ozgur et al. 2015; Zeeshan et al. 2016). ER stress can also lead to ROS production, and this can also occur subsequent to accumulation of unfolded protein in the ER (Bhandary et al. 2012; Ozgur et al. 2015; Zeeshan et al. 2016). One study revealed that GSH and GSSG may be good markers of oxidative stress in the blood and other tissues, as GSSG accumulates and the GSH/GSSG ratio decreases under conditions of oxidative stress (Giustarini et al. 2013). However, data regarding the role of ER stress and UPR effectors in varicocele are scarce. In the current study, we revealed an important relationship between ROS-mediated ER stress and varicocele. We investigated the ability of ROS to evoke ER stress in a varicocele-induced model and found that the UPR effectors GRP-78, p-IRE1α and p-JNK were triggered in a ROS-dependent manner.

MOTILIPERM was previously reported to ameliorate ROS-induced infertility in a rat model of cisplatin-induced infertility (Soni et al. 2015). In the present study, the oxidative stress levels were significantly higher in all varicocele-induced groups compared to the other groups. In contrast, the MDA level in the VC + M 200 group was significantly lower than in the VC + vehicle and VC + M 100 groups. Our results reveal that varicocele exerts adverse effects on the testes, which may be attenuated by MOTILIPERM.

There are three signalling pathways initiated by ER stress sensors (Kadowaki and Nishitoh 2013): activating transcription factor-6 (ATF6), protein kinase RNA-like endoplasmic reticulum kinase (PERK) and inositol-requiring transmembrane kinase/endoribonuclease 1 (IRE1). The IRE1 pathway is activated by prolonged ER stress. The IRE1 complex may lead to activation of the JNK pathway. ER stress-induced activation of the JNK pathway may in turn trigger apoptosis (Nishitoh et al. 2002). In the current study, we conclude that MOTILIPERM was able to affect redox rebalancing with decreased activation of the UPR pathway and related proteins, such as GRP-78, p-IRE1α and p-JNK. Therefore, our observations support a close interplay between oxidative stress and ER dysfunction. These data suggest that MOTILIPERM suppresses ROS-mediated ER stress in varicocele.

## Conclusions

Our results show a connection between sensitizer-induced redox imbalance and establishment of ER-stress-induced injury in the varicocele-induced rat model. For the first time, we report ROS-mediated activation of p-IRE1α and p-JNK and apparent rescue by MOTILIPERM in the varicocele rat model. MOTILIPERM can decrease varicocele-related infertility caused by ROS-dependent ER stress and UPR.
